# Case of Cephalalgia, in Which the Sulphate of Quinine Was Employed Successfully

**Published:** 1824-03

**Authors:** Thomas Bushell

**Affiliations:** London.


ART. II.—*Case of Cephalalgia, in which the Sulphate of Quinine was employed successfully.* By THOMAS BUSHELL, M.R.C.S. London. (Communicated through Dr. JAMES JOHNSON.)

THOMAS SHELDRAKE, ætat. forty-one, had been butler in a family in York-place nearly sixteen years. His mode of living had always been regular and abstemious. His disposition appeared to be of the sombre cast, but still he was accustomed to much activity. In the month of October, 1822, the affection about to be described came on, and for its cure he had consulted three or four of our most eminent practitioners, under whose superintendence he had been several times relieved; but his disorder had always returned, within a few days, with undiminished violence. I had seen him, during a great part of this time, frequently, but not professionally: it had, however, struck me most forcibly, from his appearance and hurried manner, that he must be labouring under a great degree of nervous irritability.

About the middle of June, 1823, he first applied to me for assistance. His symptoms were severe (I might say, excruciating,) pain in the head, coming on in the evening, continuing during the night, and preventing sleep altogether, but gradually receding towards morning. (Not unfrequently, however, the pain would last for days together without any remission.) This was accompanied by occasional acute stabs, or darts, from one side of the head to the other, which struck down the upper part of the spine, and in the direction of the inferior maxillary nerve, bearing some affinity to tic doloureux, or neuralgia. During the severity of the paroxysms, his eyes would be suffused with tears and ferretty, his face flushed, and scalp tender to the touch; at times I have observed him, with his hands applied to the sides of his head, in excessive torture. In the absence of the paroxysm his countenance was pale. His pulse was generally below eighty, small, and feeble; tongue foul, and requiring scraping in the morning; appetite very bad; bowels irregular, and motions dark and offensive.

The treatment appeared obvious: tonic aperients and alterative mercurial doses had soon the best effect, and Mr. S. expressed himself as quite well. This relief was but of short duration; in three or four days the whole train of symptoms returned with increased energy. A repetition of the same mode of treatment was unsuccessful. Cupping largely, blistering, purges, and salines, tended only to injure my patient's general health, without in the least alleviating the encephaloid symptoms.

August.—Dr. Ley now saw him, and prescribed tonic aperients; to which, in a few days, was added the pulv. colchici, in five-grain doses. This produced a surprising change, and my patient again considered himself as well, and Dr. Ley took his leave; but in a very few days, although persisting in the use of medicine, all was as bad as ever.

I now tried cinchona, valerian, and the medicines called nervines; but they were quite useless.

From the symptoms partaking at times so much of the character of a distinct neuralgic affection, I determined on the trial of the ferri subcarbonas; but this seemed, after a few days, to augment the severity of the paroxysms of cerebral erethism.

September.—Dr. Warren now saw him, and strongly recommended the cinchona; but Sheldrake, finding that all his medical advisers were disposed to adopt the same plan, and having taken the bark several times before without benefit, wished to decline taking any more medicine. I prevailed on him to take the sulphate of quinine, and I administered to him about twenty doses of one grain each, ter die, with improvement. He now left town for Sandgate, where however he staid but a few days, and returned in a worse state, if possible, than ever.

I now resolved to give the ferri subcarbonas a full trial: he took it but a few days, and again left town for Sandgate. The disease here, however, increased, and Mr. S. Hutchinson, of Hythe, brother of Mr. H. of Southwell, was called in to him. Mr. H., in his letter to me, states that he "was desired to see him in the evening of October 21st, at which time he was easier from what was described as having been a more serious and severe attack of the peculiar affection of head under which he labours, than any which had been before witnessed. I listened attentively to the description given by himself and others of this and his former attacks; and, though the disease was in some degree anomalous, yet the idea of neuralgia occurred most forcibly to my mind, and I determined on giving the ferri subcarbonas. On the following evening, I saw him during the time he was suffering pain, and found him with a very full and bounding pulse, a flushed face, and hot skin. I immediately took from his arm, in a full stream, upwards of twenty ounces of blood, and he expressed himself relieved. This blood, on the next morning, was more highly inflamed, cupped, and buffy, than any I had ever seen in the most acute attacks of inflammatory disease. I, in consequence, suspended the use of the ferrum, gave him strong purgative pills, antimonials, and salines, and repeated the venesection on the two following days. He had a large blister on the back of his neck, and his bowels were kept in a constant state of action until the 29th; when, the pulse being natural, the pain trifling in comparison, though still continuing so as to make him fear a more serious relapse, I again commenced the use of the carbonate of iron, in doses of ℈j. ter die, with gr. v. of the pulv. rhei. The doses were gradually increased, and he now takes ℈ij, ter die, I should say, with manifest good effect."

November 9th.—He again came under my care, certainly improved, and I continued, at the suggestion of Mr. Hutchinson, the ferrum, augmenting the dose, with the occasional interposition of an aperient. It did not, however, appear to me to be doing as much good as could be wished. His tongue continued foul, his bowels irregular, and countenance most miserable. In about a fortnight, his former violent symptoms were threatening a return.

December 1st.—His prospect was now a melancholy one: he seemed to be no forwarder than he was the first day I prescribed for him. I now adopted a purgative plan of treatment, which was always productive of a certain degree of relief.

20th.—No advantage being obtained, I resolved on trying to a full extent the sulphate of quinine; he commenced with one grain and a half doses, in the form of pill, every three hours.

21st.—Augment the dose to two grains tertiis horis.

22d.—This medicine was evidently more beneficial than any thing he had before taken. His head was much better, his tongue cleaner, and appetite on the increase. The dose was augmented to three grains, and religiously taken at the third hour.

23d.—On the evening of this day, I found him extremely active in his occupation; and he said he believed he was quite well. The medicine was exerting its full influence on his system; he was in complete broil, perspiring most copiously; his countenance was flushed and heated, and his pulse like a bell-rope: in fact, he was labouring under a high degree of excitement. His head, however, was free from every sort of uneasiness. I directed the medicine to be taken at longer intervals.

January 1st, 1824.—Quite well; his appetite not to be complained of, his nights good, and gaining flesh.

6th.—Says "he shall do now," being better than for the last sixteen months; his bowels act without the aid of medicine, and his tongue natural.

9th.—Has a severe cold, but no visit of his former enemy.

January 18th.—Continued well, excepting his catarrh, till the 16th, when he had threatenings of his malady. He passed two sleepless nights, and on the succeeding morning, when walking out, he was seized with violent pain darting from the orbit, in the direction of the frontal twig of the ophthalmic branch of the par trigeminum, and also the same sort of pain which seemed to emanate from the foramen maxillare anticum. This continued about a quarter of an hour, and he stated that, had it lasted much longer, he must have gone mad.

22d.—This day he is as well as he ever was in his life, and attributes his unpleasant feelings on the 16th and 17th to the effects of his cold.

31st.—Continues in every respect well.

In reviewing my notes on this case, I am drawn into a few observations as to whence the *fons et erigo mali?* Could the whole train of symptoms take their source in the derangements of the gastric functions? Was it from sympathy with that morbid action of the splanchnic and other nerves, which had induced the depraved hepatic and chylopoietic secretions; or is it possible that undigested and fermenting aliment can, by irritation of these nervous tissues, which appear destined for other purposes rather than common sensibility, produce a state of derangement, which shall only manifest itself in their cerebral or spinal basis? If such is the case, we have before us a malady with all the grades from simple head-ach, or a slight erethismal state of the brain, to nearly cerebral inflammation itself. Occasionally I could only imagine that some morbid action must be taking place, either in the brain or its meninges, which would terminate in alteration of structure, or a state of disease which would deprive my patient of his existence or mental faculties. Bleeding and other evacuants produced but little effect on these states: the patient's immediate sufferings were certainly mitigated, but no advance was gained by their use in preventing the recurrence of the same violent symptoms. At one time the strictly antiphlogistic treatment, at another the tonic, would appear to be advantageous, but there was no dependence on either. During the intermission of the paroxysms of Mr. S.'s malady, the digestive organs were prominent features of ailment, and the remedies calculated to obviate their unhealthy actions were those which exerted most influence over the cephalalgic symptoms. Several times the alvine evacuations approached to the natural state, the tongue cleaned, and the appetite mended, and as often the head improved; but something was yet wanting to completely correct the still vitiated state of the chylopoietic juices. Can it be allowed that the free application of the sulphate of quinine to the guardians of these secretions was a sufficient stimulus to correct the particular state of irritation under which they had so long laboured. I am disposed to allow the remedy all this; for under its use the bowels became regular, the secretions natural, and my patient free from all his bad symptoms.

Unquestionably, the salifiable basis of the cinchona, by itself, or in combination with acids properly prepared, will be a medicine of great service. The sulphate of quinine appears to excite, from this and some other trials I have made with it, a most potent influence on the system, and, by the different accounts we receive from the continent, may be taken to a very considerable extent without proving deleterious, like many of our other active remedies. In the present state of our knowledge of its powers, I hope the above case may not be without its use in introducing it to the attention of the profession in similar anomalous affections.

